# Structured Logic: A Philosophical Framework for Diagnostic Imaging Studies

**DOI:** 10.7759/cureus.86957

**Published:** 2025-06-29

**Authors:** Kevin Rivera, Sam S Ahn

**Affiliations:** 1 Emergency Medicine, The Ohio State University Wexner Medical Center, Columbus, USA; 2 Surgery, Texas Christian University, Fort Worth, USA

**Keywords:** diagnostic imaging risks, fluoroscopy intervention, interdisciplinary teaching, philosophy, x ray

## Abstract

Philosophical frameworks are rarely made explicit in clinical research, yet they underlie decisions at every stage of study design. This technical report presents a case example of how a logical positivist epistemology, which emphasized observable data, inductive generalization, and methodological disinterestedness, was intentionally applied to a vascular imaging study involving duplex ultrasound and venography in the diagnosis of pelvic vein compression syndromes. While the focus is on imaging, the relevance of epistemological transparency extends broadly to clinical trials, diagnostic tests, and AI-driven decision support. The study's design, data abstraction, and analytic processes were grounded in a transparent philosophical foundation, as documented in the author’s original thesis. This report outlines the methodological structure, defends the choice of positivism, compares competing frameworks, and reflects on how epistemology can shape research validity.

## Introduction

While empirical rigor is widely discussed in clinical research, the philosophical foundations that guide research design are often unacknowledged. However, defining one's epistemological stance, which is how knowledge is acquired and validated, is especially important in fields like medical imaging, where diagnostic inferences rely on probabilistic associations and interdisciplinary interpretation [1,2]. More broadly, this concern applies to diagnostic testing, clinical trials, and AI implementation across medical specialties.

Medical imaging operates within complex clinical systems that integrate anatomic, physiological, and computational knowledge, making it essential to clarify the assumptions underlying the interpretation of data [2]. This technical report presents a case-based application of formal epistemological principles, derived from research on early detection of May-Thurner Syndrome (MTS) using duplex ultrasound and venography. The study was designed using a logical positivist approach, as defined in Ryan's framework of research paradigms, the philosophy of biology, and Novikov and Novikov's schema for philosophical-methodological alignment [1-4]. This methodological design was first developed and implemented in the author's original thesis [5].

## Technical report

Epistemological justification and framework selection

The study was grounded in logical positivism, which emphasizes empirical observation, testable hypotheses, and operationalized variables [1,3]. Positivism was selected for its alignment with imaging-based research, which depends on observable, reproducible data. While positivism has been critiqued for overlooking unmeasurable phenomena, its strength lies in ensuring objectivity, which is critical in retrospective image-based investigations [3]. This choice was deliberate, especially given the retrospective nature of the study and the need for reproducibility.

Alternative philosophical paradigms such as Bayesianism and Popper's falsifiability-based post-positivism were considered. Bayesianism, while powerful in clinical reasoning and machine learning, often integrates subjective prior beliefs, which can conflict with the disinterestedness sought in this study [4,6]. Post-positivism allows for theoretical constructs but risks overinterpretation when variables are not directly measurable [4,6]. Table 1 presents a comparison of these frameworks.

**Table 1:** Common Epistemological Frameworks in Clinical Research

Framework	Basic Definition	Typical Use Cases	Strengths	Limitations
Logical Positivism	Knowledge is derived from observable, measurable phenomena	Imaging studies, structured registries, AI model input design	High reproducibility, objectivity	Ignores non-measurable constructs, undervalues context
Bayesianism	Belief updating based on prior probability and new data	Clinical reasoning, AI/ML, predictive modeling	Integrates prior knowledge, flexible	Sensitive to bias in priors, requires assumptions
Post-Positivism	Science progresses by falsifying hypotheses; knowledge is provisional	Hypothesis-driven trials, policy research	Embraces uncertainty, allows theories	Harder to operationalize, allows more interpretation

Structured data collection and methodological design

All imaging data were obtained retrospectively and de-identified. Data abstraction was limited to variables that could be directly observed and consistently measured: percent stenosis, waveform morphology, and reflux patterns. Variables were not inferred or theorized; instead, findings were categorized using objective criteria predefined in the imaging reports [5]. The abstraction process began by isolating all cases that had undergone intravascular ultrasound (IVUS). Duplex ultrasound and venography results that preceded IVUS in these patients were abstracted based on predefined measurable parameters: percent compression at the iliac vein, presence of continuous vs. phasic flow, reflux duration, and collateral formation. All abstractions were performed blinded to IVUS results to minimize bias, consistent with Merton’s principle of disinterestedness [4].

Inductive reasoning and interpretive boundaries

Inductive reasoning was used to draw probabilistic associations between earlier imaging modalities and the diagnostic reference standard. Logistic regression was employed to identify associations while acknowledging that such findings are inherently probabilistic, especially when generalizing from a clinical cohort [5]. The study accepted the limitation that certainty cannot be derived from correlation alone, reinforcing the epistemological position that inference in retrospective imaging must remain grounded in observable trends [7].

Translational potential and interdisciplinary relevance

This framework aligns with contemporary thinking on translational science, which emphasizes integrated methods to answer multifaceted clinical questions [8]. This is important in imaging settings where AI tools are increasingly used. Ensuring that AI training data are grounded in observable, reproducible imaging features protects against inappropriate generalizations and enhances transparency in validation [9]. Positivist framing here complements algorithmic logic, which depends on defined input variables rather than interpretive reasoning. The relevance of epistemological clarity extends to registries, diagnostic studies, and clinical trial design.

## Discussion

Philosophical framing in clinical research

This case illustrates that explicit epistemological framing is not only feasible but beneficial in imaging-based clinical research. Grounding the study in logical positivism ensured that every component, from the selection of observable variables to the neutral structure of data abstraction, was aligned with an overarching commitment to empirical reproducibility. Transparency about inductive reasoning clarified the boundaries of inference, reinforcing that correlations in retrospective imaging do not imply causality.

Practical relevance can be seen in how philosophical framing helps avoid interpretive overreach. For example, in our own May-Thurner dataset, disinterested abstraction of compression percentages and waveform patterns helped prevent post-hoc reinterpretation once IVUS results were known. In contrast, prior studies have suffered from overinterpretation of venographic reflux without structured abstraction criteria, leading to highly variable diagnostic thresholds. Similarly, AI systems trained on unstructured or theory-laden radiologic variables may embed biases that go undetected until real-world failures occur, as documented in radiology AI applications evaluated by Pesapane et al. [9]. Table 2 compares transparent and implicit epistemological framing in different steps during study design.

**Table 2:** Comparing Transparent vs Implicit Epistemological Framing in Study Design

Study Feature	Philosophically Transparent	Implicit Assumptions
Variable selection	Explicitly defined, observable	May include proxies or theory-laden variables
Abstraction process	Blinded, reproducible criteria	Informal or outcome-informed abstraction
Inference method	Inductive, with probabilistic bounds	Unstated or suggestive of causation
Bias control	Based on epistemic norms (e.g., disinterestedness)	Based on tradition or convenience

Practical implementation guide

1. Begin each study design by explicitly stating your epistemological stance. 2. Select variables that align with that stance (e.g., observable for positivism). 3. Use abstraction protocols that limit interpretive bias. 4. Define how inferences will be drawn (e.g., probabilistically, not deterministically). 5. Revisit your philosophical assumptions during interpretation to avoid overreach.

Limitations

While logical positivism promotes clarity and objectivity, its application is not without limitations. Positivist frameworks can exclude meaningful phenomena that are difficult to observe directly, such as pain experience or subjective patient outcomes. In the context of vascular imaging, variables like patient positioning, clinical symptoms, and longitudinal symptom relief may fall outside its scope. Overcommitment to positivist constraints could underrepresent contextual nuance or dismiss valuable hypothesis-generating observations. Furthermore, generalization from retrospective data, even when grounded in positivist principles, cannot eliminate confounding or historical biases inherent to chart-based research.

## Conclusions

Clinical research that explicitly states and applies its philosophical assumptions can enhance transparency, reproducibility, and interdisciplinary communication. This technical report demonstrates how a logical positivist framework applied rigorously to a vascular imaging study served as a methodological compass from study design to data interpretation. By aligning methods with a clear epistemic foundation, the study gained clarity and interpretive strength. Future imaging research would benefit from adopting similar philosophical transparency, particularly in retrospective or diagnostic contexts that rely heavily on inference and pattern recognition.

This model can be extended to retrospective studies in other domains where imaging findings are central to clinical inference. It also offers a structure for designing imaging registries with philosophically grounded variable definitions and for guiding the development of artificial intelligence based on sound principles of inference. Finally, embedding this framework into research education may help future investigators develop more transparent, reproducible, and conceptually coherent studies in diagnostic science.
